# Coherent terahertz control of metastable magnetization in FePS_3_

**DOI:** 10.1038/s41467-026-78164-x

**Published:** 2026-10-06

**Authors:** Batyr Ilyas, Tianchuang Luo, Honglie Ning, Emil Viñas Boström, Alexander von Hoegen, Jaena Park, Junghyun Kim, Je-Geun Park, Angel Rubio, Nuh Gedik

**Affiliations:** 1 Department of Physics, Massachusetts Institute of Technology, Cambridge, MA USA; 2 Max Planck Institute for the Structure and Dynamics of Matter, Hamburg, Germany; 3 Nano-Bio Spectroscopy Group, Departamento de Física de Materiales, Universidad del País Vasco, San Sebastián, Spain; 4 Department of Physics and Astronomy, Seoul National University, Seoul, Republic of Korea; 5 Center for Quantum Materials, Seoul National University, Seoul, Republic of Korea; 6 Institute of Applied Physics, Seoul National University, Seoul, Republic of Korea; 7 Initiative for Computational Catalysis, The Flatiron Institute, New York, NY USA

**Keywords:** Magnetic properties and materials, Phase transitions and critical phenomena

## Abstract

Driving specific lattice modes with ultrafast stimuli offers a dynamic route for tailoring material properties out of equilibrium. However, achieving dynamic coherent control of the nonequilibrium phases via resonant excitation of lattice coherences remains largely unexplored. In this study, we demonstrate coherent phononic control of a newly discovered, light-induced metastable magnetization in the van der Waals antiferromagnet FePS_3_. By using a sequence of terahertz (THz) pulses, we modulate the magnetization amplitude at the frequencies of phonon coherences, whose infrared-active nature and symmetries are further revealed by polarization- and field-strength-dependent measurements. Furthermore, our two-dimensional THz spectroscopy, in tandem with first-principles numerical simulations, shows that these phonons nonlinearly displace a Raman-active phonon, which induces the metastable net magnetization. These findings not only clarify the microscopic mechanism underlying the metastable state in FePS_3_ but also establish vibrational coherences in solids as a powerful tool for ultrafast quantum phase control, enabling manipulation of material functionalities far from equilibrium.

## Abstract (web summary)

Light-driven phonons offer a route to nonequilibrium phases, yet coherent, on demand control of such phases has been hard to achieve. Here, Ilyas, Luo, Ning and co-authors show that the timing of two terahertz pulses sets the amplitude of a metastable magnetization in FePS_3_, through anharmonic coupling of infrared active phonons to a magnetization inducing Raman mode.

## Introduction

The crystal structure is a key factor in determining the emergent phenomena in quantum materials. Control of lattice arrangements has proven to be a powerful route to discovering new phases. For instance, in two-dimensional layered materials, the introduction of long-wavelength superlattice periodicity through twisting or lattice mismatch has unveiled a wide variety of correlated electronic orders, including superconductivity^1,2^ and fractional quantum anomalous Hall states^3–5^. In magnetic systems, changes in the stacking configuration of atomic layers can similarly lead to diverse magnetic ground states^6,7^. More broadly, lattice tuning through established methods such as external pressure^8,9^ and epitaxial strain^10,11^ has long been effective in stabilizing phases inaccessible under ambient conditions. Beyond these static approaches, dynamic structural control through selective excitation of phonon resonances has recently emerged as a promising approach to inducing novel nonequilibrium phases^12,13^. This technique has enabled the realization of symmetry-broken phases that either have no equilibrium analogs^14–16^ or are stabilized at temperatures exceeding their thermodynamic limits^17,18^. Specifically, the concept of dynamical control of magnetism via coherent lattice excitations has been established by recent pioneering works^17,19–24^. This rapidly evolving direction of phase manipulation holds great promise for ultrafast and energy-efficient functional applications.

Despite these advances, many aspects of phononic control protocols remain poorly understood. One significant yet underutilized advantage is that phonons exhibit substantially longer coherence times than electrons^25,26^, which opens up numerous possibilities for inducing, sustaining, and modifying out-of-equilibrium states. Previous studies have demonstrated coherent modulation of phase transitions through phonon modes launched by near-infrared (NIR) pulses^27,28^. However, these approaches generate substantial light-induced heating due to the excessive energy deposited into the electronic subsystem and indiscriminately excite multiple Raman-active modes of different symmetries^29^. Such heating and lack of selectivity inherently hinder the on-demand phononic manipulation of fragile, temperature-sensitive phases. To date, coherent control of phase transitions through selective and resonant excitation of phonon modes with minimized heating has not been realized.

To place this challenge in a broader context, while coherent control is well-established in molecular chemistry, its application to switch entire macroscopic quantum phases in solid-state systems is fundamentally distinct and challenging. Unlike isolated molecules, solids possess a continuum of electronic and phononic excitations that act as a thermal bath, typically inducing decoherence. However, if such an environment can be properly navigated, crystalline solids may provide unique advantages. For example, using resonant terahertz excitation to isolate a specific, phase-preserving lattice pathway can extend coherent control far beyond the transient femtosecond timescales accessible in molecules. Demonstrating that such a coherence survives long enough to deterministically dictate a macroscopic order parameter represents a fundamental leap from local molecular manipulation to the long-lived dynamic control of quantum materials.

Achieving this functionality requires meeting two conditions. First, an infrared (IR)-active phonon, resonantly excited with minimal disturbance to the electronic subsystem, must be driven by a tailored sequence of THz pulses. These pulses enable controlled constructive or destructive modulation of their amplitude. Second, this mode must couple nonlinearly and strongly to a Raman-active phonon that ultimately modifies the physical properties. Here, we realize this strategy in FePS_3_, demonstrating the coherent control of a hidden metastable magnetic state via a double-THz-pulse excitation scheme. While prior observations^16^ established the existence of this THz-induced state, achieving coherent control over it was not an anticipated outcome. If the phase transition were driven by a conventional Raman process proportional to the square of the driving field, coherent control would be inefficient and limited to the brief duration of the THz pulses. Instead, using two-dimensional THz spectroscopy, we prove that the deterministic switching of the magnetization is governed by a two-phonon ionic pathway. Finding this specific mechanism provides the necessary physical framework to explain how macroscopic magnetic order can be controlled over extended timescales, moving beyond the observation of light-induced states to their active manipulation.

### Principles of coherent control of magnetization in FePS_3_

**Fig. 1:**
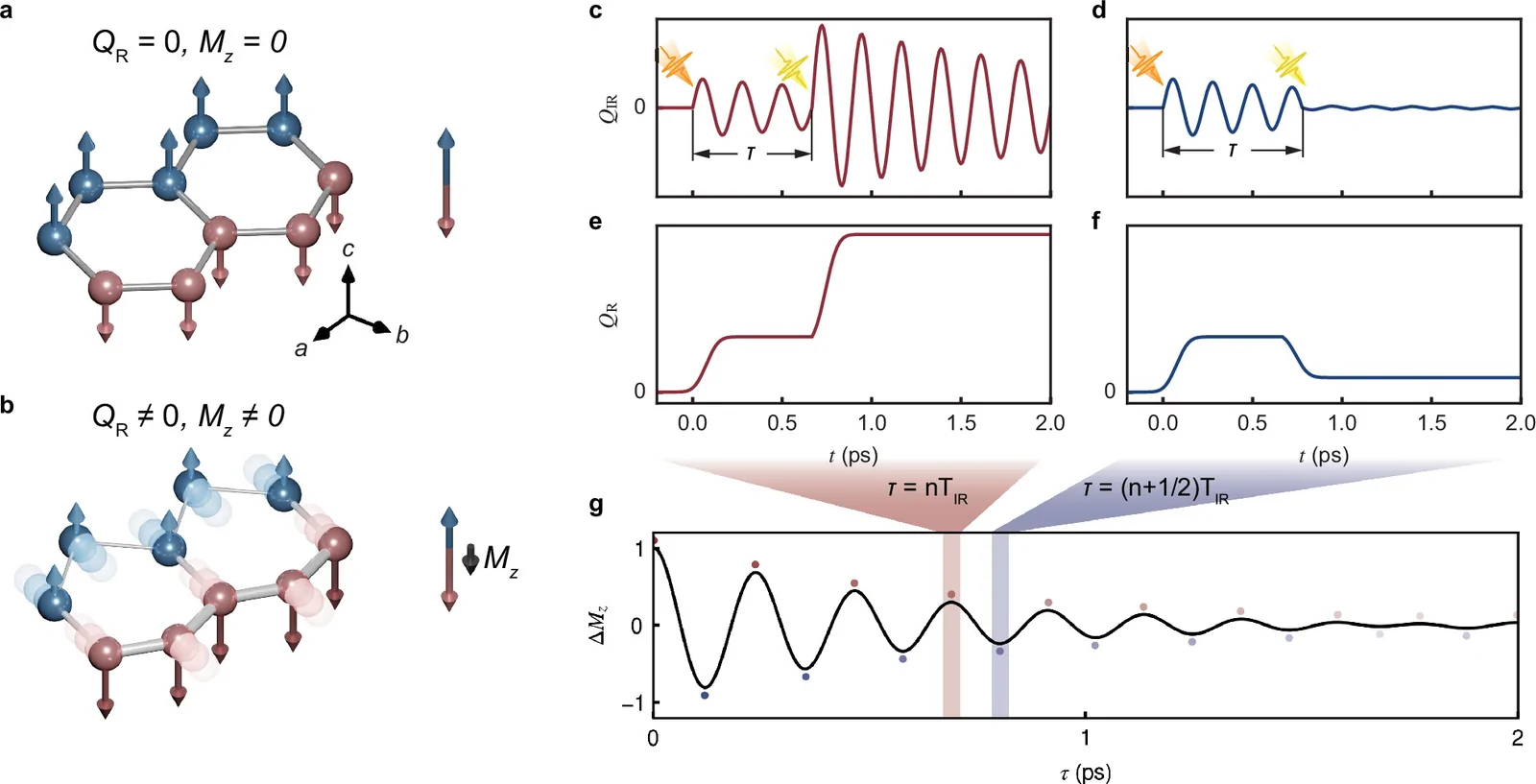
Principle of coherent phonon control of the metastable magnetization. **a** Equilibrium crystal and magnetic structure of FePS_3_, with red and blue arrows indicating Fe spins pointing up and down in the out-of-plane direction, respectively. The right panel shows the fully compensated up and down spins. **b** Crystal lattice displaced along the *Ω*_R_ = 3.27 THz Raman phonon, showing enhanced exchange interactions within the red zigzag chain (thick bonds) and weakened exchange interactions within the blue zigzag chain (thin bonds). The right panel illustrates uncompensated up and down spins, generating net magnetization, *M*_*z*_. **c**, **d** Schematics of IR phonon amplitude as a function of time in the double-pump experiment for *τ* = *nT*_IR_ (**c**), and $\tau=(n+\frac{1}{2}){T}_{{{{\rm{IR}}}}}$ (**d**), demonstrating coherent enhancement (**c**) or suppression (**d**). **e**,** f** Corresponding Raman phonon displacement as a function of time resulting from IR phonon driving. **g** Schematics of the THz-induced magnetization as a function of THz pulse separation *τ*, with red and blue shadings corresponding to *τ* = *nT*_IR_ and $\tau=(n+\frac{1}{2}){T}_{{{{\rm{IR}}}}}$, respectively.

FePS_3_ exhibits exceptionally strong spin-lattice coupling among van der Waals (vdW) magnets^30–35^, making it an ideal platform for exploring the control of magnetic order through structural tuning. The magnetic Fe^2+^ ions are arranged in a hexagonal lattice, with spins forming anti-aligned zigzag ferromagnetic chains. Within this structure, spins point out of the plane due to strong magnetic anisotropy, resulting in Ising-like magnetic behavior^36^ (Fig. 1a). Previous results have demonstrated that displacing the lattice along a specific Raman-active phonon mode at *Ω*_R_ = 3.27 THz strengthens the nearest-neighbor magnetic bonds within one zigzag chain, while weakening interactions in the adjacent chain^16^. Such modulation thereby alters the strength of magnetic exchange interactions in neighboring zigzag chains in an opposite way, leading to the emergence of a net magnetization out of the antiferromagnetic (AFM) motif (Fig. 1b). Although the incident THz pulse preserves pure time-reversal symmetry (${{{\mathcal{T}}}}$) and the macroscopic AFM ground state preserves the combined spatio-magnetic equilibrium symmetry (${{{{\mathcal{T}}}}}^{{\prime} }={{{\mathcal{T}}}}\tau$, where *τ* is a lattice translation, see Supplementary Note 7), a net magnetization emerges through a dynamic effect^21^. Specifically, the THz-driven Raman structural distortion dynamically breaks ${{{{\mathcal{T}}}}}^{{\prime} }$, achieving a specific symmetry breaking that cannot be replicated by any macroscopic static strain or stress. Finally, while the initial structural drive decays within picoseconds, the induced magnetization persists for milliseconds: the spin relaxation is heavily suppressed by critical slowing down near the Néel temperature^16^, and the lattice adiabatically follows this slow spin dynamics (Supplementary Note 8).

We can coherently control the metastable magnetization amplitude via three steps. First, by adjusting the timing between two nearly identical excitation pulses, the amplitude of an IR-active phonon can be significantly enhanced or suppressed. Specifically, when the time delay between pulses is an integer multiple of the phonon period, the forces from both pulses constructively interfere, amplifying the phonon amplitude (Fig. 1c). Conversely, if the time delay corresponds to an odd integer multiple of half the phonon period, the forces destructively interfere, diminishing the phonon amplitude (Fig. 1d). Second, if the IR-active phonon is anharmonically coupled to the Raman-active phonon at *Ω*_R_ = 3.27 THz, this coupling induces a quasi-static distortion of the lattice along this Raman phonon eigenvector. Therefore, controlling the amplitude of the IR-active phonon will modify the displacement strength of the coupled Raman phonon (Fig. 1e, f). Third, as discussed earlier, the displacement of this specific phonon resonance in FePS_3_ leads to the emergence of a new state with net magnetization. Through the process outlined above, the magnetization amplitude will be modulated at the frequency of the IR phonon as the time delay between pulses is varied (Fig. 1g). Therefore, if experimentally realizable, coherent modulation of the IR-active phonons with resonant excitation would enable coherent control over the magnetization amplitude.

## Results

**Fig. 2:**
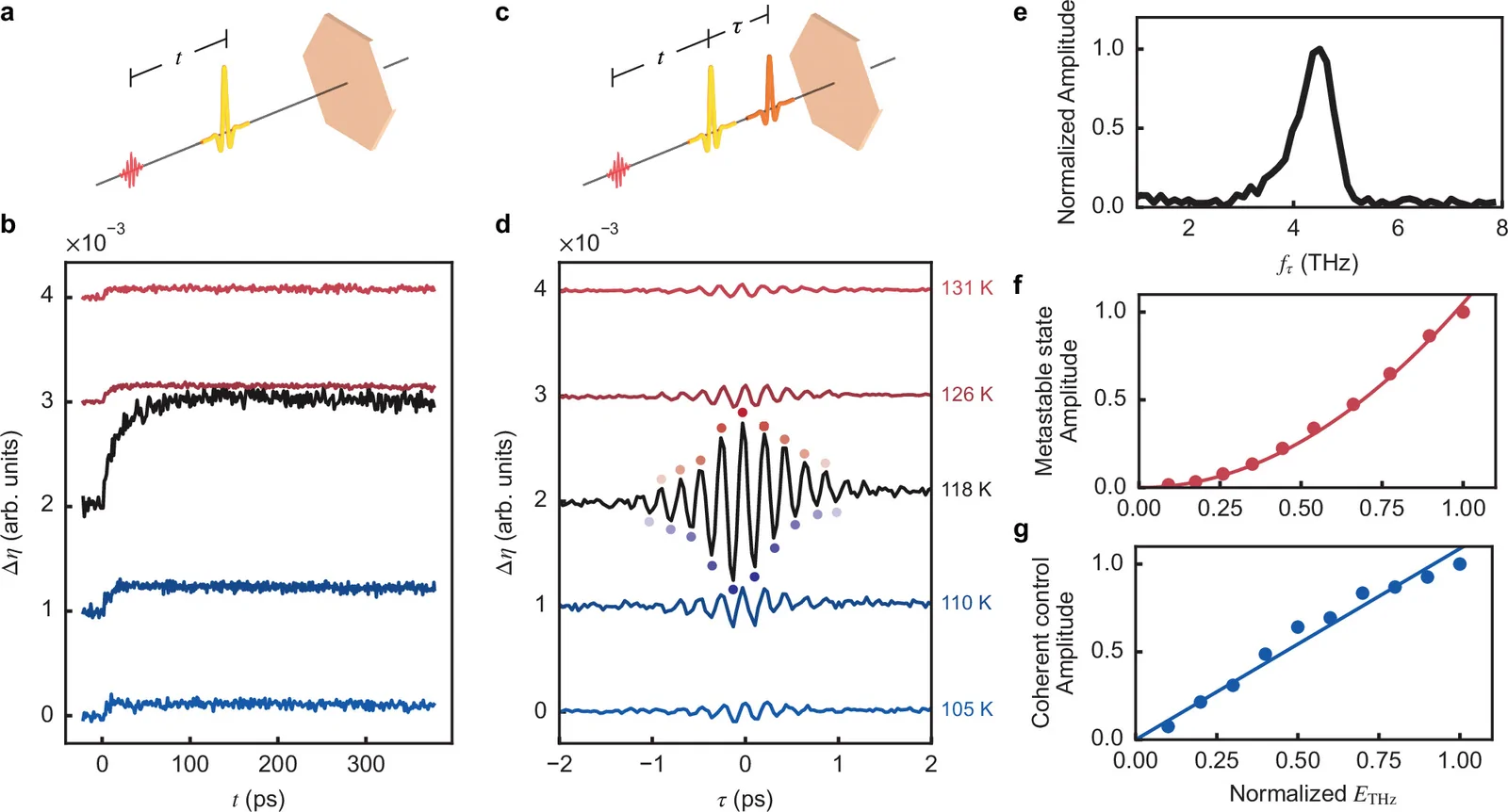
Experimental demonstration of coherent control. **a** Schematic of the single THz pump experiment, with the yellow and red pulses representing the THz pump and the 800 nm probe. **b** Ellipticity change time traces as a function of *t* at different temperatures as measured in (**a**). **c** Schematics of the double THz pump experiment, with an additional THz pulse (orange) introduced at time delay *τ*. **d** Probe ellipticity change measured at *t* = 400ps as a function of *τ* for various temperatures. **e** The Fourier transform of the *τ*-dependent trace at 118 K in (**d**), revealing a peak at 4.5 THz. **f** The metastable state magnitude in (**b**) as a function of normalized THz field strength *E*_THz_. The solid line is a quadratic fit. **g** The area of the Fourier spectrum from (**e**) as a function of normalized THz field strength of the yellow THz pulse. The solid line is a linear fit.

We probe the light-induced magnetization dynamics through the time-dependent ellipticity change of a subsequent NIR pulse in transmission geometry (Fig. 2a). Near the magnetic ordering temperature (*T*_N_ = 118 K), a single broadband THz pulse induces a new magnetic state with a net magnetization whose amplitude and lifetime increase dramatically on approaching the phase transition (Fig. 2b). Notably, this magnetization can last for milliseconds close to *T*_N_, thus forming a metastable state^16^. To assess the feasibility of coherent phononic control of magnetization, we designed a set of experiments. We first introduce a second THz pulse with a similar field profile (Fig. S1b) and continuously vary the time separation between the two pulses (*τ*) near *τ* = 0ps, while probing the induced dynamics at *t* = 400 ps when the system enters the metastable state (Fig. 2c). The material’s response exhibits coherent oscillations as a function of *τ*, with their amplitudes increasing as we approach *T*_N_ (Fig. 2d). A Fourier transform of these oscillations (Fig. 2e) reveals a broad peak near 4.5 THz, a frequency that is distinct from *Ω*_R_. Furthermore, whereas the metastable magnetization scales quadratically with the THz pulse field strength in the single-pump experiment (Fig. 2f), indicating a nonlinear excitation mechanism, the oscillation amplitude scales linearly with the electric field strength of a single THz pulse (Fig. 2g). These observations strongly suggest the involvement of an IR-active phonon that nonlinearly mediates the displacement of the Raman mode and thereby modulates the metastable magnetization. While a direct coupling between the driven infrared phonon and the macroscopic magnetization might intuitively seem possible, the Ginzburg-Landau analysis shows that this direct pathway cannot produce the critical enhancement near *T*_N_. The nonlinear rectification of the infrared drive into the Raman-active mode is therefore the essential mechanism responsible for the long-lived magnetization observed experimentally (see Supplementary Note 6).

**Fig. 3:**
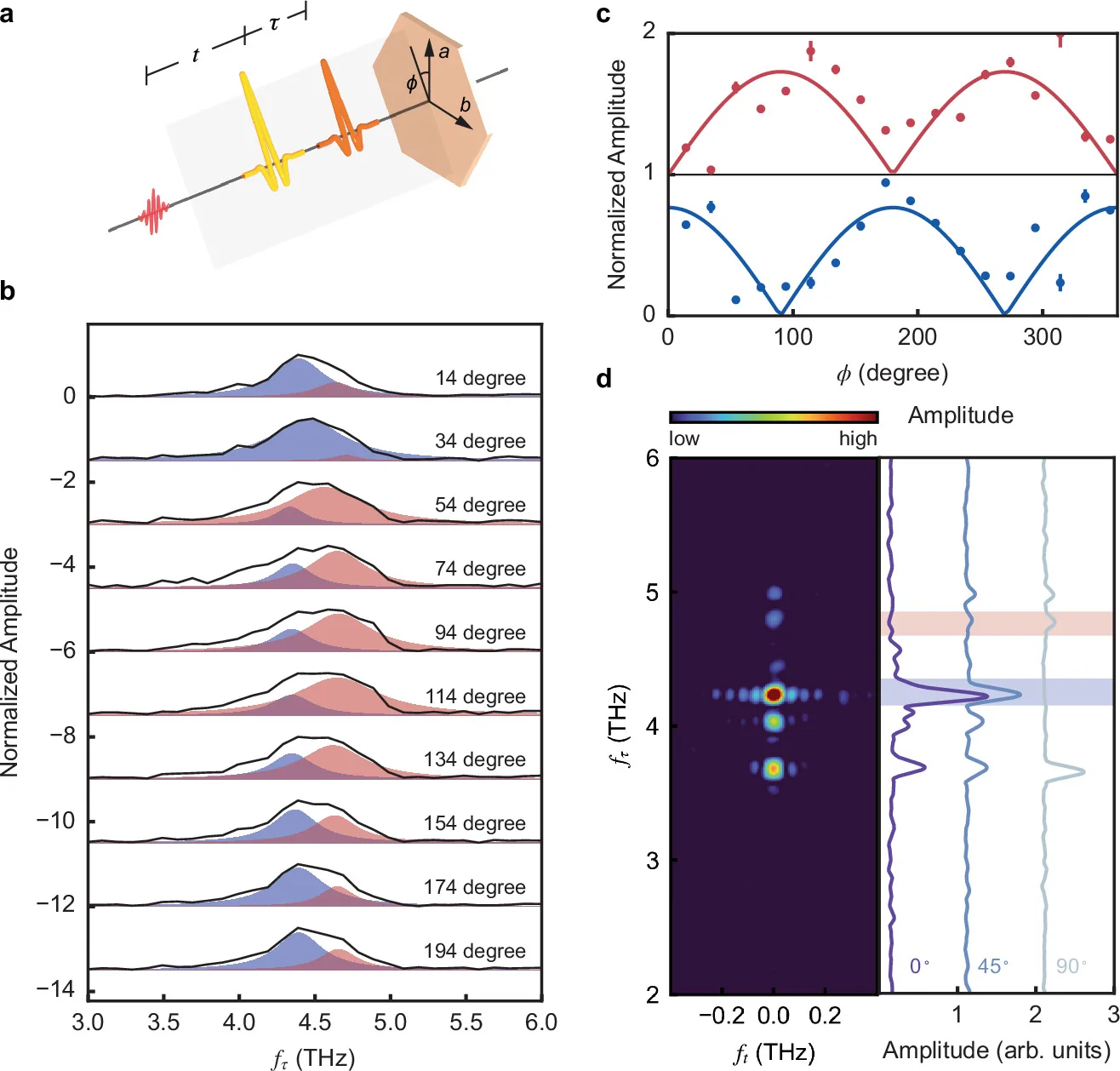
Identification of the IR-active phonons mediating the lattice displacement. **a** Schematic of the THz polarization dependence experiments, where the THz pulses are co-rotated. *ϕ* denotes the angle between THz polarization and the crystallographic *a*-axis. **b** Evolution of the oscillation spectrum in Fig. 2e with *ϕ*. The blue and red shaded areas are Lorentzian oscillator fits with Eq. S7. **c** The amplitude of the two Lorentzian oscillators as a function of *ϕ*, with $| \sin \phi |$ (red solid line) and $| \cos \phi |$ (blue solid line) fits demonstrating orthogonal polarizations. Data and fits are vertically offset for clarity. **d** Left: 2D THz spectrum near *f*_*t*_ = 0 THz obtained at *T* = 10 K and *ϕ* = 45^∘^. Right: Linecuts along *f*_*t*_ = 0 THz of 2D THz spectra measured at *ϕ* = 0^∘^, 45^∘^, 90^∘^. The blue and red shaded regions correspond to the blue and red oscillator frequencies in (**b**, **c**).

We further examine the nature of the IR-active mode by studying the evolution of the oscillations as we simultaneously alter the polarization angle of the two THz pulses (Fig. 3a). This polarimetry measurement allows us to determine the symmetry of the IR mode(s)^37,38^, necessary for further elucidating their anharmonic interaction with the *Ω*_R_ Raman-active mode. The Fourier spectra show a transfer of spectral weight between two frequency positions around 4.3 and 4.7 THz as we rotate the THz polarization (Fig. 3b). The intensities of these two peaks maximize along orthogonal directions, implying the presence of two IR-active modes with similar energies and mutually perpendicular dipole moments. Indeed, the broad peak near 4.5 THz can be fit using two Lorentzians given in Eq. S7, whose weights change with polarization in an out-of-phase fashion (Fig. 3c). These measurements indicate that the low- and high-frequency IR-active phonons carry electric dipole moments along the crystallographic *a* and *b* axes, and therefore follow *B*_u_ and *A*_u_ symmetries, respectively.

To investigate more closely whether the IR phonon indeed mediates the displacement of the *Ω*_R_ Raman mode, we performed two-dimensional THz spectroscopy (2DTS) measurements, a powerful technique for directly probing the nonlinear interactions between low-energy excitations and rectification processes^37,39,40^ (see also Fig. S3). We conducted these measurements at *T* = 10 K, where the larger phonon amplitudes and longer coherence times, compared with *T* ≈ *T*_N_, allow for better examination of their interactions (see Fig. S4 for comparison). We continuously varied both the inter-pump time separation (*τ*) and the pump-probe delay (*t*), whereas *t* is now scanned over a short range (−1 ps < *t* < 10 ps) with fine steps to resolve different modes (see Supplementary Note 4 and Fig. S3). Such two-dimensional time scans (*τ* and *t*) are converted into two-dimensional frequency maps (*f*_*τ*_ and *f*_*t*_) via a Fourier transform (Fig. 3d, Left). The displacement of the Raman mode is manifested in the 2D spectrum as the rectification signals near *f*_*t*_ = 0 THz. As shown in Fig. 3d, two strong spectral weight puddles appear near *f*_*τ*_ = 4.5 THz, in agreement with the frequencies extracted from the polarimetry measurements in Fig. 3b, c. These observations provide evidence for the involvement of the two nearly degenerate infrared phonons at 4.5 THz in displacing the lattice and in dictating the coherent control of magnetization.

We repeated the 2DTS measurements at different polarization angles of both THz pulses to further elucidate the symmetry of the infrared modes displacing the lattice. The time traces and the corresponding frequency-frequency maps are provided in Fig. S3. The vertical linecuts at *f*_*t*_ = 0 THz (Fig. 3d, right) reveal the dominance of the mode near *f*_*τ*_ = 4.3 THz (4.7 THz) when the electric field is oriented along the crystallographic *a*-axis (*b*-axis), suggesting its *B*_u_ (*A*_u_) symmetry, in agreement with the results shown in Fig. 3b, c. We note that the linewidths of these modes at *T* = 10 K are much smaller than those near *T* = 118 K, allowing for better resolution of the broad spectral peak.

### Nonlinear phonon interactions

The pathway connecting the IR-active phonons to the THz-induced magnetization is revealed by considering a theoretical model that includes all possible nonlinear couplings. We model the dynamics of the IR and Raman modes as coupled harmonic oscillators. The IR phonon *Q*_IR_ is directly driven by the electric field of the THz pulse. In contrast, the Raman phonon *Q*_R_ can be driven by three different mechanisms up to the second order in the electric field. First, the Raman phonon can be quadratically driven by the light field with the force proportional to ${E}_{\,{\mbox{THz}}\,}^{2}$^41^, where *E*_THz_ represents the experimentally measured THz field. Second, known as ionic Raman scattering, it can be launched exclusively through nonlinear interactions with IR-active phonons, with the corresponding driving force proportional to ${Q}_{\,{\mbox{IR}}\,}^{2}$^42,43^. The third possible mechanism, referred to as infrared resonant Raman scattering, involves both an IR phonon and the THz field^44,45^, with the force proportional to *E*_THz_*Q*_IR_. The latter two scenarios, involving the IR modes, are candidates for our coherent phononic control protocol.

**Fig. 4:**
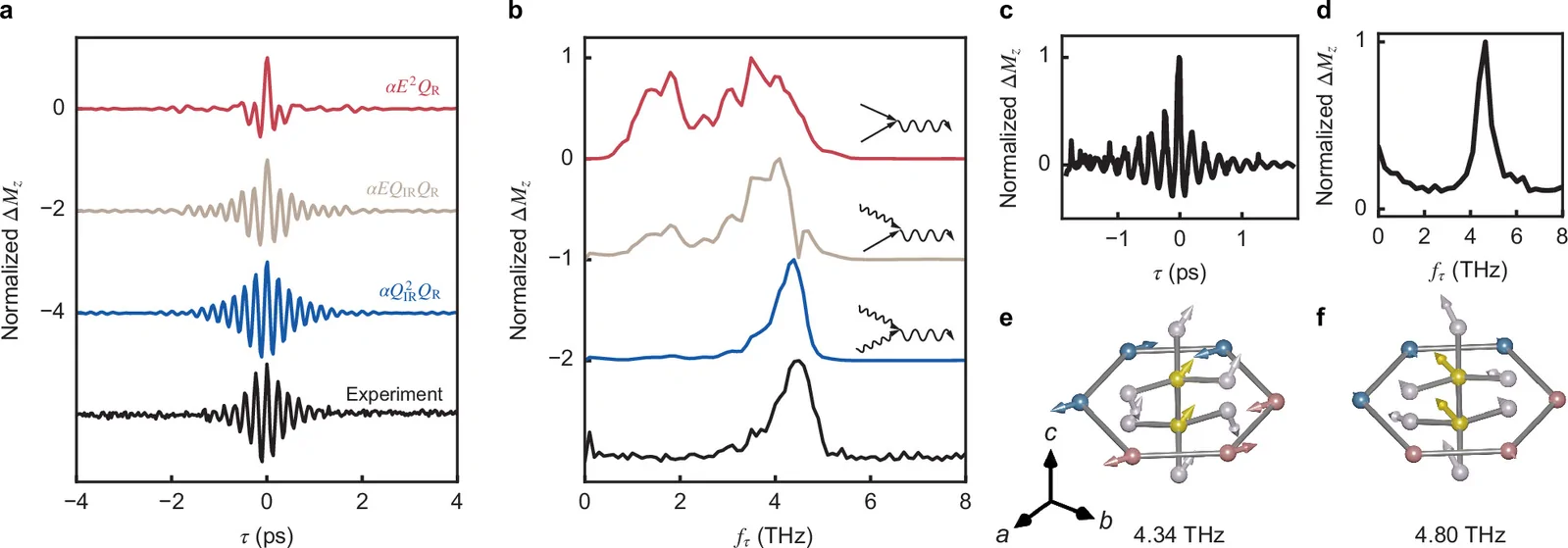
Possible nonlinear interaction pathways. **a** Simulated Δ*M*_*z*_(*τ*) when two-photon excitation (red), infrared resonant Raman scattering (beige), and ionic Raman scattering (blue) mechanisms are activated for driving the Raman mode *Ω*_R_. The experimental result is shown in black. **b** Fourier transform of the time traces in (**a**). The inset shows the diagram of the nonlinear interactions, where the straight arrows represent photons and the wavy arrows represent phonons. **c** Atomistic spin dynamics simulation of Δ*M*_*z*_(*τ*) with the ionic Raman scattering mechanism activated. **d** Fourier transform of (**c**). **e**,** f** Schematics of the calculated IR phonon eigenmodes at 4.34 and 4.80 THz. The red and blue spheres represent Fe ions with spins pointing in opposite directions. The yellow and white spheres represent phosphorus and sulfur atoms, respectively.

The magnetization *M*_*z*_ is modeled by overdamped dynamics, driven by the Raman mode through the coupling term *gQ*_R_*LM*_*z*_^16,46^, where *M*_*z*_ is the magnetization magnitude, *L* is the Néel vector magnitude, and *g* is the coupling constant. We then derive the equations of motion for each variable (Eq. S1) and numerically solve the dynamics of the IR phonon, the Raman phonon, and the magnetization by including two THz pulses matching the experimental configuration (see Eq. S2). Next, we analyze the magnetization amplitude as we vary the time delay between THz pulses, *τ*, while setting the probe pulse at a large delay time of *t* = 400 ps. The simulation results in the time domain for each aforementioned scenario are provided in Fig. 4a, with their Fourier transform shown in Fig. 4b. Remarkably, only the ionic Raman scattering channel closely reproduces the experimental data, indicating the dominance of the anharmonic phonon coupling in inducing the metastable magnetized state.

While the above phenomenological model captures the essence of our experimental observations, we further performed atomistic spin dynamics simulations to account for critical fluctuations near *T*_N_. Using a 20 × 20 supercell with the Fe^2+^ spins initialized in thermal equilibrium through Monte Carlo simulated annealing (see Supplementary Note 2), we modeled the double-pump coherent control experiment with the ionic Raman scattering pathway activated. The resulting THz-induced magnetization *M*_*z*_(*τ*) (Fig. 4c, d) shows quantitative agreement with both the phenomenological model (Fig. 4a, b, blue curve) and the experimental data (Fig. 4a, b, black curve). Furthermore, first-principles phonon calculations in FePS_3_ identify two IR-active phonon modes: a 4.34 THz mode with *B*_u_ symmetry (Fig. 4e) and a 4.80 THz mode with *A*_u_ symmetry (Fig. 4f). Their frequencies and corresponding symmetries closely align with our experimental assignments (Fig. 3b–d), further validating our interpretation.

## Discussion

In summary, we have demonstrated coherent control of the nonequilibrium metastable magnetized phase in FePS_3_ using resonant THz pulses. Our results extend the principles of coherent control in the field of femtochemistry^47,48^ from molecular systems to crystalline materials. Notably, additional comparative double-pump experiments with near-infrared pulses (Fig. S5) showed no coherent modulation, underscoring the unique advantages of the THz approach. This remarkable selectivity of THz radiation for specific phononic and magnonic degrees of freedom promises access to a wide range of nonequilibrium phases of matter. While the specific *gQ*_R_*LM*_*z*_ coupling and zigzag magnetic order are unique to FePS_3_, the underlying control mechanism is broadly applicable. Rectifying an infrared phonon into a Raman distortion alters local bond geometries. Because magnetic exchange interactions strongly depend on these structural parameters, this nonlinear phononic approach provides a general pathway to coherently control magnetic states in other systems with strong spin-lattice coupling. Practically, the ability to switch magnetization on and off with THz pulses effectively functions as an ultrafast quantum gate, enabling on-demand control with picosecond-scale response time that is orders of magnitude faster than conventional gigahertz quantum gates^49,50^. Looking ahead, leveraging materials with longer phonon coherence times, we may transform this platform into quantum memory systems, combining laser-based switching and magnetic readout. Our work thus establishes a fundamental framework for coherent terahertz manipulation of quantum materials, with promising implications for information processing technologies through precise control of emergent phases.

## Methods

### Sample preparation

FePS_3_ single crystals were synthesized from iron (Sigma-Aldrich, 99.99% purity), phosphorus (Sigma-Aldrich, 99.99%), and sulfur (Sigma-Aldrich, 99.998%) using the chemical vapor transport method. The powdered elements were prepared inside an argon-filled glove box. The starting materials were mixed in the stoichiometric ratio, with an additional 5 wt% of sulfur to compensate for its high vapor pressure. We verified the stoichiometry of the synthesized single crystals with a COXEM-EM30 scanning electron microscope equipped with a Bruker QUANTAX 70 energy-dispersive X-ray system. The crystal structure was confirmed through X-ray diffraction measurements using a commercial diffractometer (Rigaku Miniflex II). Prior to experiments, single crystals were freshly cleaved to obtain clean surfaces.

### Double-THz-pump experiments

The experimental schematic of our double-THz-pump measurements is illustrated in Fig. S1a. The 800 nm output of a 1 kHz Ti:sapphire amplifier is split into two beams. The stronger beam pumps an optical parametric amplifier (OPA), whose signal and idler wavelengths are set to 1450 and 1785 nm, respectively. These beams are modulated using optical choppers at half (500 Hz) and quarter (250 Hz) of the laser repetition rate to facilitate the detection of nonlinear signals through lock-in techniques^40,51^. The temporal delay between the idler and the signal pulses is controlled by a mechanical delay stage (DS2). Both the signal and idler beams generate intense THz fields in an N-benzyl-2-methyl-4-nitroaniline (BNA) crystal through the optical rectification process. The generated THz radiation is collected and focused onto the sample with a set of three gold-coated parabolic mirrors (PM1-3). The weaker 800 nm beam, delayed from the signal beam by DS1, is focused onto the sample, overlapping with the THz beams. The THz-induced polarization ellipticity change of the transmitted probe beam is measured using the standard balanced detection technique. The time traces of the THz pulses generated by idler (*E*_1_) and signal (*E*_2_) are shown in Fig. S1b, with the corresponding spectra shown in Fig. S1c. Both THz pulses exhibit a broad spectrum spanning 0–6 THz.

### First-principles calculations

To obtain the phonon modes of FePS_3_, we performed first-principles simulations with the abinit electronic structure code^52–55^. We used the local density approximation with projector augmented wave (PAW) pseudopotentials, plane-wave cut-offs of 20 Ha and 40 Ha for the plane-wave and PAW parts, respectively, and included an empirical Hubbard *U* of 2.7 eV on the Fe *d*-orbitals as self-consistently determined in the Octopus electronic structure code via the ACBN0 functional. A Γ-centered Monkhorst-Pack grid with dimensions 8 × 6 × 8 was used to sample the Brillouin zone. The ground state was found to have zigzag antiferromagnetic order with spins aligned along the *z*-axis.

Phonon frequencies and eigenvectors were calculated with ABINIT after relaxing the atomic positions and stresses to below 10^−6^ Ha/Bohr. We find a relevant Raman phonon mode at *Ω*_R_ = 3.27 THz, as well as several IR-active modes in the range 4−5 THz. Out of the possible IR modes, we identify a *B*_u_ mode at 4.34 THz and an *A*_u_ mode at 4.80 THz, in good agreement with our experimental data.

## Supplementary information

Supplementary Information

Transparent Peer Review file

## Data Availability

Datasets collected and/or analyzed during the current study are available on the Harvard Dataverse at https://doi.org/10.7910/DVN/HF911O^56^.
