# Supplementary material for: Coherent terahertz control of metastable magnetization in FePS3
Source: Nat Commun. 2026 Oct 6;17:11387. doi: 10.1038/s41467-026-78164-x (PMC13736905; doi:10.1038/s41467-026-78164-x)
Supplement: Supplementary file 1 [file 41467_2026_78164_MOESM1_ESM.pdf]

Supplementary Materials for ”Coherent terahertz control of metastable magnetization in $FePS_3$”

Batyr Ilyas[1,†], Tianchuang Luo[1,†], Honglie Ning[1,†], Emil Viñas Boström[2,3], Alexander von Hoegen[1], Jaena Park[4,5], Junghyun Kim[4,5], Je-Geun Park[4,5,6], Angel Rubio[2,3,7], Nuh Gedik[1,*]

[1]Department of Physics, Massachusetts Institute of Technology, Cambridge, MA, 02139, USA.

[2]Max Planck Institute for the Structure and Dynamics of Matter, Hamburg, 22761, Germany.

[3]Nano-Bio Spectroscopy Group, Departamento de Fisica de Materiales, Universidad del Pais Vasco, San Sebastian, 20018, Spain.

[4]Department of Physics and Astronomy, Seoul National University, Seoul, 08826, Republic of Korea.

[5]Center for Quantum Materials, Seoul National University, Seoul, 08826, Republic of Korea.

[6]Institute of Applied Physics, Seoul National University,Seoul, 08826, Republic of Korea.

[7] Initiative for Computational Catalysis, The Flatiron Institute, New York, NY, 10010, USA.

[*]Corresponding author. Email: gedik@mit.edu [†]These authors contributed equally to this work.

Supplementary Notes

1 Numerical simulation of coupled mode dynamics in double-THz-pump experiments

To elucidate the dominant nonlinear coupling mechanism that drives the metastable state, we developed numerical simulations assuming that the IR phonon and Raman phonon are two driven oscillators and the light-induced magnetization exhibits an overdamped exponential decay. These three channels are coupled abided by symmetry and are described by three coupled differential equations:

$$\begin{aligned}
\frac{d^2Q_{\mathrm{IR}}}{dt^2} + 2\Gamma_{\mathrm{IR}}\frac{dQ_{\mathrm{IR}}}{dt} + \omega_{\mathrm{IR}}^2 Q_{\mathrm{IR}} &= Z^* E + 2\alpha_{11} Q_{\mathrm{IR}} Q_{\mathrm{R}}, \\
\frac{d^2Q_{\mathrm{R}}}{dt^2} + 2\Gamma_{\mathrm{R}}\frac{dQ_{\mathrm{R}}}{dt} + \omega_{\mathrm{R}}^2 Q_{\mathrm{R}} &= \alpha_{00} E^2 + \alpha_{01} E Q_{\mathrm{IR}} + \alpha_{11} Q_{\mathrm{IR}}^2, \\
\frac{dM_z}{dt} &= \Gamma_M(-2M_z + gQ_{\mathrm{R}}).
\end{aligned} \tag{1}$$

Here, Q_{IR} represents the IR-active mode with eigen-frequency $\omega_{\mathrm{IR}} = 2\pi(4.5\ \mathrm{THz})$ and damping coefficient $\Gamma_{\mathrm{IR}} = 2$ THz. Q_{R} denotes the Raman-active mode with $\omega_{\mathrm{R}} = 2\pi(3.3\ \mathrm{THz})$ and damping coefficient $\Gamma_{\mathrm{R}} = 20$ THz. These values are all acquired from the experiment. Only one of the IR-active modes is included in the simulation for simplicity. Z^* is the effective charge, which reflects the oscillator strength of Q_{IR}. α_{00}, α_{01}, and α_{11} are the excitation strengths of Q_{R} in the two-photon, one-photon-one-phonon, and two-phonon excitation channels. M_z is the sample magnetization along the c-axis, which evolves with a very small phenomenological relaxation rate $\Gamma_M = 10^{-4}$ THz. Finally, g is the coefficient of the coarse-grained spin-phonon coupling term $gQ_{\mathrm{R}}LM_z$. As the value of the antiferromagnetic order parameter L remains largely unchanged, its dynamics are ignored for simplicity.

The driving field E is adopted from the experimentally determined THz field time traces E_1 and E_2 as depicted in Fig. 1b:

$$E(t) = E_1(t+\tau) + E_2(t). \tag{2}$$

The temporal evolution of Q_{IR}, Q_{R}, and M_z over t is computed numerically with a 4th-order Runge-Kutta method over the time range $-3\ \mathrm{ps} < t < 200\ \mathrm{ps}$ and intra-pump delay $-5\ \mathrm{ps} < \tau < 5\ \mathrm{ps}$ to emulate the experiment. Additional simulations are run with E_1 and E_2 set to zero, respectively, to

obtain the nonlinear coherent modulation component (*1*)

$$\Delta M_z(t,\tau) = M_z^{E_1,E_2}(t,\tau) - M_z^{E_1=0,E_2}(t,\tau) - M_z^{E_1,E_2=0}(t,\tau). \tag{3}$$

Finally, we plot the saturation value of ΔM_z at large t ($\Delta M_z(t = 400$ ps)) as a function of τ, which is shown in Fig. 2d.

2 Monte Carlo simulations and atomistic spin dynamics

To supplement the Ginzburg-Landau dynamics, we performed real time simulations of the atomistic spin system. For this purpose we consider the spin-phonon Hamiltonian

$$\begin{aligned} H = &\sum_{ij} \mathbf{S}_i \cdot (\mathbf{J}_{ij}\mathbf{S}_j) - \Delta \sum_i (S_i^z)^2 + \frac{1}{2}\sum_i \left[P_i^2 + \Omega_i^2 Q_i^2\right] + \gamma Q_\mathrm{R} \sum_{ij} A_{ij}\mathbf{S}_i \cdot \mathbf{S}_j \\ &+ g Q_\mathrm{R} Q_\mathrm{IR}^2 + Z E(t) Q_\mathrm{IR}, \end{aligned} \tag{4}$$

including two phonon modes at 3.27 THz and 4.5 THz (the Raman active mode and one of the IR-active modes) and the external THz electric field $E(t)$. The 4.5 THz mode is considered to be linearly driven, with a light-matter coupling given by the Born effective charge Z. The Hamiltonian leads to the following coupled equations of motion

$$\begin{aligned} \frac{\partial \mathbf{S}_i}{\partial t} &= \mathbf{S}_i \times \frac{\partial H}{\partial \mathbf{S}_i} \\ \frac{\partial^2 Q_\mathrm{R}}{\partial t^2} &= -\Omega_\mathrm{R}^2 Q_\mathrm{R} - g Q_\mathrm{IR}^2 - \gamma \sum_{ij} A_{ij}\mathbf{S}_i \cdot \mathbf{S}_j \\ \frac{\partial^2 Q_\mathrm{IR}}{\partial t^2} &= -\Omega_\mathrm{IR}^2 Q_\mathrm{IR} - 2g Q_\mathrm{R} Q_\mathrm{IR} + Z E(t). \end{aligned} \tag{5}$$

For the electric field we use the experimental pulses, separated by a delay τ.

The equilibrium state of the magnetic system at a temperature $T = T_\mathrm{N}$ was prepared with simulated annealing using the Monte Carlo Metropolis algorithm. All exchange parameters have been calculated from first principles, and are given in Ref. (*2*). The calculations were performed for a monolayer sample of $FePS_3$ using a spin length $S = 2$ and an in-plane supercell of linear size $L = 20$ unit cells. The simulated annealing was initialized at a temperature $T \approx 170$ K and performed down to a target temperature $T = T_\mathrm{N} = 115$ K in steps of $\Delta T \approx 2$ K. At each

temperature we performed 2000 Monte Carlo sweeps to thermalize the system, followed by 4000 measurements performed at an interval of 50 sweeps to obtained thermodynamic averages. Our calculations find a magnetic transition temperature in good agreement with experiment, thereby validating the calculated equilibrium spin parameters.

At the transition temperature, the statistical average of the magnetization, $M_z = \langle \sum_i S_i^z \rangle$, is found to vanish. To investigate its dynamics, we then apply the THz field $E(t) = E_1(t+\tau) + E_2(t)$ assuming a Born effective charge $Z = 8$ (from 4 Fe ions each with an effective charge $Z_0 = 2$) and a phonon-phonon coupling $g = 1$ meV. The time evolution was performed up to a maximal time $t = 662$ ps, using $N_t = 20000$ time steps and a step size of $dt = 0.033$ ps. The magnetization was evaluated as a function of τ, at a time $t_{\mathrm{eval}} = 200$ ps after the pulse E_2, when the magnetization has reached a saturated value. The resulting magnetization is shown in Fig. 4c, and its Fourier transform in Fig. 4d.

3 Polarization dependence of the coherent control of metastable states

The modulation of the metastable state as a function of time delay τ between the double THz pulses for various THz polarizations is shown in Fig. 2. The THz polarization of both E_1 and E_2 are simultaneously rotated from $\phi = 14°$ to $\phi = 374°$ with $20°$ increments. Their corresponding spectra as shown in Fig. 2b and Fig. 3b is obtained through Fourier transforming the time traces in Fig. 2a. The transformed spectra are then fitted with two complex Lorentzian functions (*3*), because a coherent oscillation can be modeled as a damped harmonic oscillator with maximum amplitude at $\tau = 0$:

$$f(\tau) = A\cos(2\pi f_0 \tau + \varphi)e^{-\gamma|\tau|}, \tag{6}$$

where A represents the maximum amplitude, f_0 is the oscillation frequency, ϕ denotes the oscillation phase, and γ is the coherence damping rate. The corresponding Fourier transform of Eq. 6 is given by the addition of two Lorentzians

$$\tilde{f}(\omega) = A\gamma \left[\frac{e^{i\varphi}}{\gamma^2 + (\omega - 2\pi f_0)^2} + \frac{e^{-i\varphi}}{\gamma^2 + (\omega + 2\pi f_0)^2} \right]. \tag{7}$$

Since our analysis focuses exclusively on the positive frequency domain, we can neglect the second term in Eq. 7 and therefore the Fourier spectrum of each IR phonon can be modeled by a Lorentzian. The polarization-dependent amplitude of the two modes (Fig. 3c) is extracted by fitting both the real and imaginary parts of the spectrum with a sum of two oscillators described by Eq. 7. The fitted results are shown in Fig. 2b.

4 2D THz spectroscopy of the coherent modes in $FePS_3$

The 2D THz responses of $FePS_3$ as a function of (t, τ) measured at 10 K are presented in Fig. 3. The measurements are performed at various THz polarization angles ϕ. To obtain the frequency-domain spectra shown in Fig. 3d, we perform a 2D Fourier transform over the region bounded by the black dashed lines in the upper-right quadrant of the time-domain data. This region is selected to maximize the signal-to-noise ratio while avoiding temporal artifacts from the pump-pump and pump-probe overlap region.

The complete 2D THz spectra are presented in Fig. 3d-f. The horizontal axis (f_t) represents the detection frequency, while the vertical axis (f_τ) corresponds to the excitation frequency. The sharp peaks observed in these spectra can be classified into the following categories (*4*):

1. **Nonlinear coupling peaks ($f_\tau \neq f_t \neq 0$):** These off-diagonal peaks indicate nonlinear coupling between IR-active phonons at frequencies f_τ and Raman-active phonons at frequencies f_t. They can originate from either two-phonon or one-phonon-one-photon processes.

2. **THz rectification peaks ($f_t = 0$):** They represent displacements generated from two-phonon or two-magnon nonlinearities.

3. **Magnon nonlinear peaks** ($f_t = f_M$ or $f_\tau = f_M$. $f_M = 3.7$ THz is the magnon frequency)**:** These peaks, which include $(f_t, f_\tau) = (f_M, f_M), (f_M, 0), (0, f_M), (2f_M, f_M)$ arise due to magnon anharmonicities.

Notably, THz rectification peaks at $f_t = 4.2$ THz and 4.8 THz are observed (Fig. 3d), indicating their role in creating lattice displacement as discussed in the main text.

5 Absence of coherent modulation with double NIR pumps in $FePS_3$

To demonstrate that THz pulses are crucial for coherent control of the metastable state, we carried out double-pump experiments with photon energies in the near-infrared (NIR) range in $FePS_3$ at 115 K. First, we pump $FePS_3$ with a single NIR pulse at 1450 nm. Ultrafast demagnetization is detected in the polarization rotation channel $\Delta\theta$ (Fig. 5a). We then added a second NIR pulse at 1785 nm and scanned the time delay between the two NIR pump τ with t fixed to 370 ps. Only a sharp enhancement of the signal is observed when the two pumps overlap, indicating the absence of coherent modulation with NIR pumps (Fig. 5b).

6 Analysis of direct infrared phonon-induced magnetization

In this section, we consider the possibility of a direct coupling between the infrared (IR) phonons and the magnetic order based on effective Ginzburg-Landau theory.

We evaluate the direct coupling between infrared-active phonons and macroscopic magnetization within the framework of an effective Ginzburg-Landau theory, and using an effective quadratic coupling $g_{\mathrm{IR}}Q_{\mathrm{IR}}^2LM$. We assume the free energy is of the form (*2, 5*)

$$\begin{aligned} F = &\frac{a_L(T)}{2}L^2 + \frac{b_L}{4}L^4 + \frac{a_M(T)}{2}M^2 + \frac{b_M}{4}M^4 \\ &+ \frac{\Omega_{\mathrm{R}}}{2}Q_{\mathrm{R}}^2 + \frac{\Omega_{\mathrm{IR}}}{2}Q_{\mathrm{IR}}^2 + g_{\mathrm{R}}(L - \langle L\rangle)MQ_{\mathrm{R}} + g_{\mathrm{IR}}LMQ_{\mathrm{IR}}^2 + F_{\mathrm{R}}Q_{\mathrm{R}} + F_{\mathrm{IR}}Q_{\mathrm{IR}}. \end{aligned} \tag{8}$$

Here Ω_{R} and Ω_{IR} are frequencies of Raman (R) and infrared (IR) phonons. We have added the force terms F_{R} and F_{IR} coupling to the Raman and infrared phonons, for reasons that will become clear below.

To find the minima of the free energy we assume $L \gg M$ and Q, and differentiate F with respect to the later variables. This gives

$$\begin{aligned} 0 = \frac{\partial F}{\partial M} &= a_M M + b_M M^3 + g_{\mathrm{R}}(L - \langle L\rangle)Q_{\mathrm{R}} + g_{\mathrm{IR}}LQ_{\mathrm{IR}}^2 \\ 0 = \frac{\partial F}{\partial Q_{\mathrm{R}}} &= \Omega_{\mathrm{R}}Q_{\mathrm{R}} + g_{\mathrm{R}}(L - \langle L\rangle)M + F_{\mathrm{R}} \\ 0 = \frac{\partial F}{\partial Q_{\mathrm{IR}}} &= \Omega_{\mathrm{IR}}Q_{\mathrm{IR}} + 2g_{\mathrm{IR}}LMQ_{\mathrm{IR}} + F_{\mathrm{IR}}. \end{aligned} \tag{9}$$

We note that due to the quadratic coupling to $Q_{\rm IR}$, the last equation implies that $Q_{\rm IR} = 0$ for $F_{\rm IR} = 0$ (neglecting the fine tuning condition $\Omega_{\rm IR} + 2g_{\rm IR}LM = 0$). With a finite force term, we can solve both equations for the phonon coordinates to obtain

$$\begin{aligned} Q_{\rm R} &= -\frac{g_{\rm R}(L - \langle L\rangle)M}{\Omega_{\rm R}} - \frac{F_{\rm R}}{\Omega_{\rm R}} \\ Q_{\rm IR} &= -\frac{F_{\rm IR}}{\Omega_{\rm IR} + 2g_{\rm IR}LM}. \end{aligned} \tag{10}$$

We see that these couplings are of a very different form, such that phonon displacements are enhanced (suppressed) for large L for the Raman (infrared) phonon.

We now use these solutions in the equation for the magnetization, and find

$$a_M M + b_M M^3 = \frac{g_{\rm R}^2(L - \langle L\rangle)^2}{\Omega_{\rm R}} M + \frac{g_{\rm R}F_{\rm R}(L - \langle L\rangle)}{\Omega_{\rm R}} - \frac{g_{\rm IR}F_{\rm IR}^2 L}{(\Omega_{\rm IR} + 2g_{\rm IR}LM)^2}. \tag{11}$$

Considering these terms separately, we find the coupling to the Raman mode (to lowest order in $F_{\rm R}/\Omega_{\rm R}$) gives a magnetization of the form

$$M_{\rm R} = \left(\frac{g_{\rm R}F_{\rm R}}{\Omega_{\rm R}L_s}\right)\frac{\chi_L}{Va_M - (g_{\rm R}^2/\Omega_{\rm R})\chi_L} \approx \left(\frac{g_{\rm R}F_{\rm R}}{\Omega_{\rm R}a_M}\right)\frac{\chi_L}{VL_s}, \tag{12}$$

where L_s is the zig-zag saturation magnetization, and we have assumed $F_R = F_R L/L_s$ to account for the fact that Q_R is defined relative to the sign of L (*2, 5*). The dependence on the susceptibility $\chi_L = V(\langle L^2\rangle - \langle L\rangle^2) \sim |T - T_{\rm N}|^{-\gamma}$ shows the resonant response of M to any external for F_R for $T \approx T_{\rm N}$.

For the infrared phonon, we similarly get

$$b_M M_{\rm IR}^3 = -\frac{g_{\rm IR}F_{\rm IR}^2 L}{(\Omega_{\rm IR} + 2g_{\rm IR}LM_{\rm IR})^2} - a_M M_{\rm IR} \tag{13}$$

Assuming $M_{\rm IR} \ll 1$, we can linearize this equation to find

$$M_{\rm IR} = \frac{g_{\rm IR}F_{\rm IR}^2}{\Omega_{\rm IR}^2}\frac{L}{(2g_{\rm IR}^2F_{\rm IR}^2/\Omega_{\rm IR}^4)L^2 - a_M} \approx -\frac{g_{\rm IR}F_{\rm IR}^2}{\Omega_{\rm IR}^2}\frac{L}{a_M}. \tag{14}$$

Although this coupling also gives a finite magnetization for $T < T_{\rm N}$, there is no resonant enhancement close to the Néel temperature, and the effect is a factor $F_{\rm IR}/\Omega_{\rm IR}$ smaller than the Raman coupling.

Therefore, our thermodynamic modeling reveals that a direct IR pathway cannot produce the observed metastable state, making the nonlinear rectification into the Raman mode physically necessary.

We also note that the specific symmetry of the Raman phonon-induced distortion cannot be achieved by global static strain or stress. Static strain modifies the Bravais lattice vectors through an affine deformation, leading to a uniform stretching, compression, or shear of all symmetry-related bonds. By contrast, the relevant Raman mode identified in our study involves a specific internal coordinate shift where the bonds along one zig-zag chain are strengthened, while those in the adjacent chain are weakened. This staggered deformation breaks the equivalence of the antiferromagnetic sublattices and generates a net magnetization. Such alternating bond modulation cannot be generated by global uniaxial, biaxial, or hydrostatic strain, which act uniformly on the lattice.

7 Symmetry analysis of THz-induced magnetization

At first glance, the emergence of magnetization may seem counterintuitive, as both the macroscopic magnetic state of $FePS_3$ and the linearly polarized THz pulse preserve time-reversal symmetry. To clarify the magnetic symmetry of the equilibrium ground state: while the local AFM order breaks pure time-reversal symmetry ($\mathcal{T}$), the macroscopic AFM state preserves time reversal symmetry. More precisely, the ground state is invariant under the combined operation of time reversal and a lattice translation, which we denote as $\mathcal{T}' = \mathcal{T}\tau$. It is this combined symmetry that forbids a net macroscopic magnetization ($M = 0$) in equilibrium.

The emergence of a macroscopic magnetization under linearly polarized terahertz drive is a consequence of a dynamical coupling, governed by the trilinear free energy term $\mathcal{F} \propto gQ_{\mathrm{R}}LM$, where L is the AFM order parameter, M is the net magnetization, and Q_{R} is the Raman phonon displacement.

This coupling term is allowed by the equilibrium magnetic space group of $FePS_3$. Under the symmetry operation $\mathcal{T}'$:

- The AFM order parameter L is invariant by definition ($L \xrightarrow{\mathcal{T}'} L$).
- The macroscopic magnetization M is odd under time reversal but invariant under spatial translation ($M \xrightarrow{\mathcal{T}'} -M$).
- The zone-folded Raman phonon QR, which involves an out-of-phase motion between adjacent

structural layers, is invariant under time reversal but odd under the lattice translation ($Q_\mathrm{R} \xrightarrow{\mathcal{T}'} -Q_\mathrm{R}$).

Consequently, the product transforms as $Q_\mathrm{R}LM \xrightarrow{\mathcal{T}'} Q_\mathrm{R}LM$, respecting the equilibrium symmetry.

The mechanism of symmetry breaking in the driven system is as follows. The linearly polarized THz pulse displaces the lattice, and induces a finite Q_R. This lattice displacement dynamically breaks the combined $\mathcal{T}'$ symmetry of the ground state. The displaced phonon acts as an effective, internal magnetic field $H_\mathrm{eff} \propto gQ_\mathrm{R}L$, which explicitly breaks the $\mathcal{T}'$ symmetry and induces a finite macroscopic magnetization M. In this sense, the linearly polarized light does not break time-reversal symmetry directly, rather, it drives a structural distortion that dynamically breaks the spatio-magnetic symmetry ($\mathcal{T}'$) protecting the $M = 0$ state.

8 Spin-lattice relaxation dynamics near T_N

To explain the difference between the tens of picosecond duration of the lattice modes and the millisecond lifetime of the induced magnetization, in this section we outline the relevant thermodynamic and critical relaxation mechanisms. More extended analysis and simulations are provided in Ref. (*2*).

The metastable state is initiated by a THz-driven coherent phonon displacement. Time-resolved measurements show that these initial large-amplitude phonon oscillations indeed decay within tens of picoseconds (*2, 3*). However, because the structural and magnetic degrees of freedom are coupled via the trilinear free energy term $\mathcal{F} \propto gQ_\mathrm{R}LM$, the metastable magnetization M exerts a continuous force on the lattice, given by $F_\mathrm{eff} = -\frac{\partial \mathcal{F}}{\partial Q_\mathrm{R}} \propto gLM$. Consequently, the spin system maintains a small, quasi-static structural displacement for its entire duration. The lattice adiabatically follows the spin system and the millisecond lifetime is therefore dictated by the spin relaxation.

The relaxation of the spin system back to the $M = 0$ ground state is governed by critical dynamics near the Néel temperature $T_\mathrm{N} \approx 120$ K. In this regime, the magnetic correlation length ξ diverges as $\xi \sim |T - T_\mathrm{N}|^{-\nu}$. Following the theory of dynamic critical phenomena (*6*), the characteristic relaxation time scales as $\tau \sim \xi^z \sim |T - T_\mathrm{N}|^{-\nu z}$, where ν is the correlation-length exponent and z is the dynamic critical exponent. This macroscopic critical slowing down suppresses the spin

relaxation rate, extending the lifetime of the metastable magnetization into the millisecond regime, which is orders of magnitude longer than typical picosecond spin dynamics.

Finally, we note that a full ab-initio description of the phenomena discussed in this work is limited because the mechanism fundamentally depends on thermal fluctuations. Specifically, the photo-induced magnetization is only observed close to the Néel temperature, and vanishes at both higher and lower temperatures. Because first-principles methods like time-dependent density functional theory (TD-DFT) are restricted to zero temperature, they cannot capture these essential effects. In addition, simulating the long timescales needed to describe the phonon dynamics is computationally prohibitive for these methods. To explain these dynamics, we instead rely on a first-principles-based effective model (*2*), which shows that the Raman phonon at 3.27 THz mediates a coupling between the magnetization M and the fluctuations of the antiferromagnetic order L.

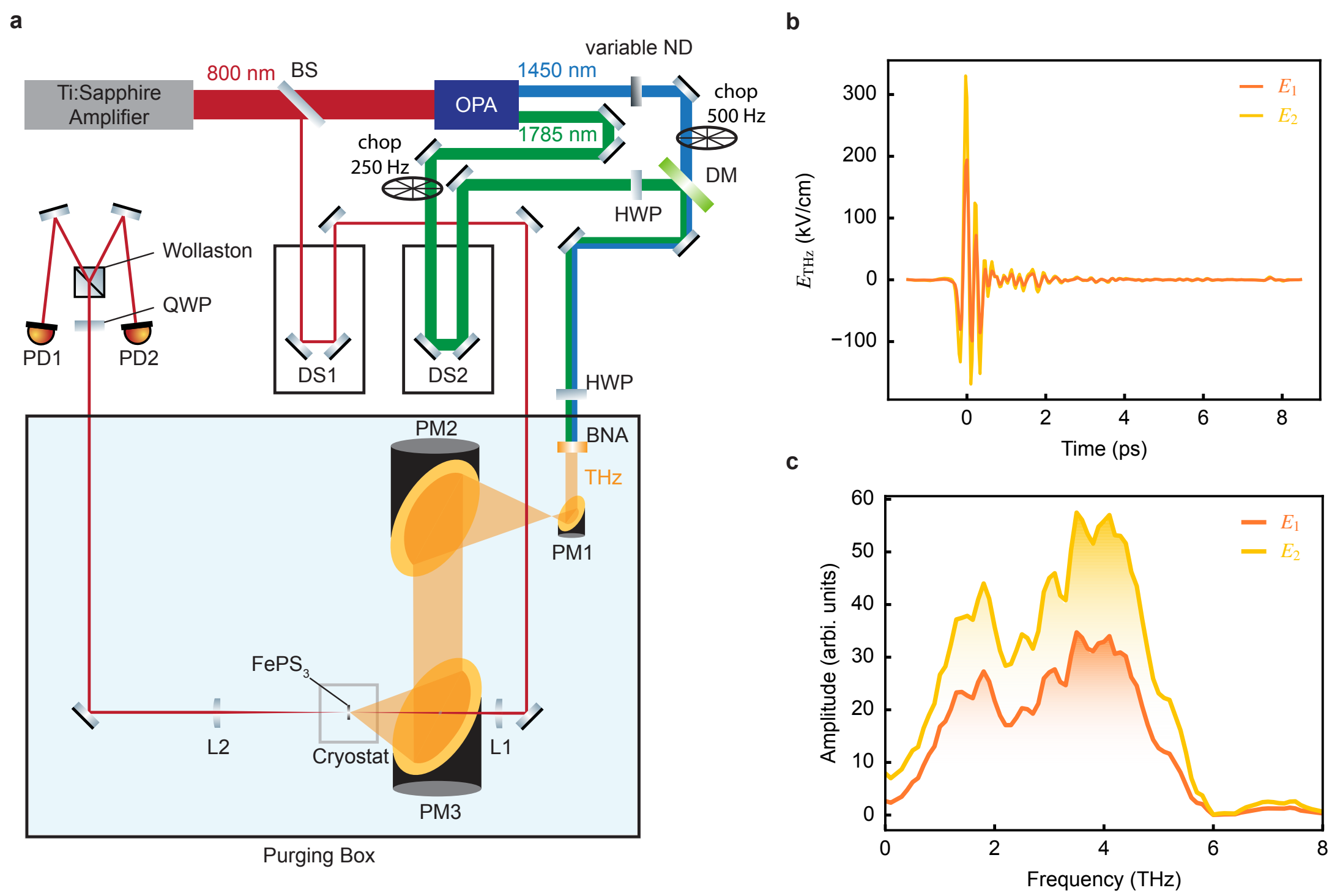

Fig. S1. **Experimental configuration and THz pulse characterization. a,** Schematic diagram of the experimental setup. BS: beam splitter, ND: neutral-density filer, DM: dichroic mirror, HWP: half-wave plate, QWP: quarter-wave plate, L: lens, PD: photodiode, DS: delay stage, Wollaston: Wollaston prism. Unlabeled optics represent mirrors. **b,** THz electric field as a function of time for E_1 (generated by 1785 nm pump) and E_2 (generated by 1450 nm pump). **c,** Fourier transform of the time traces in **b**.

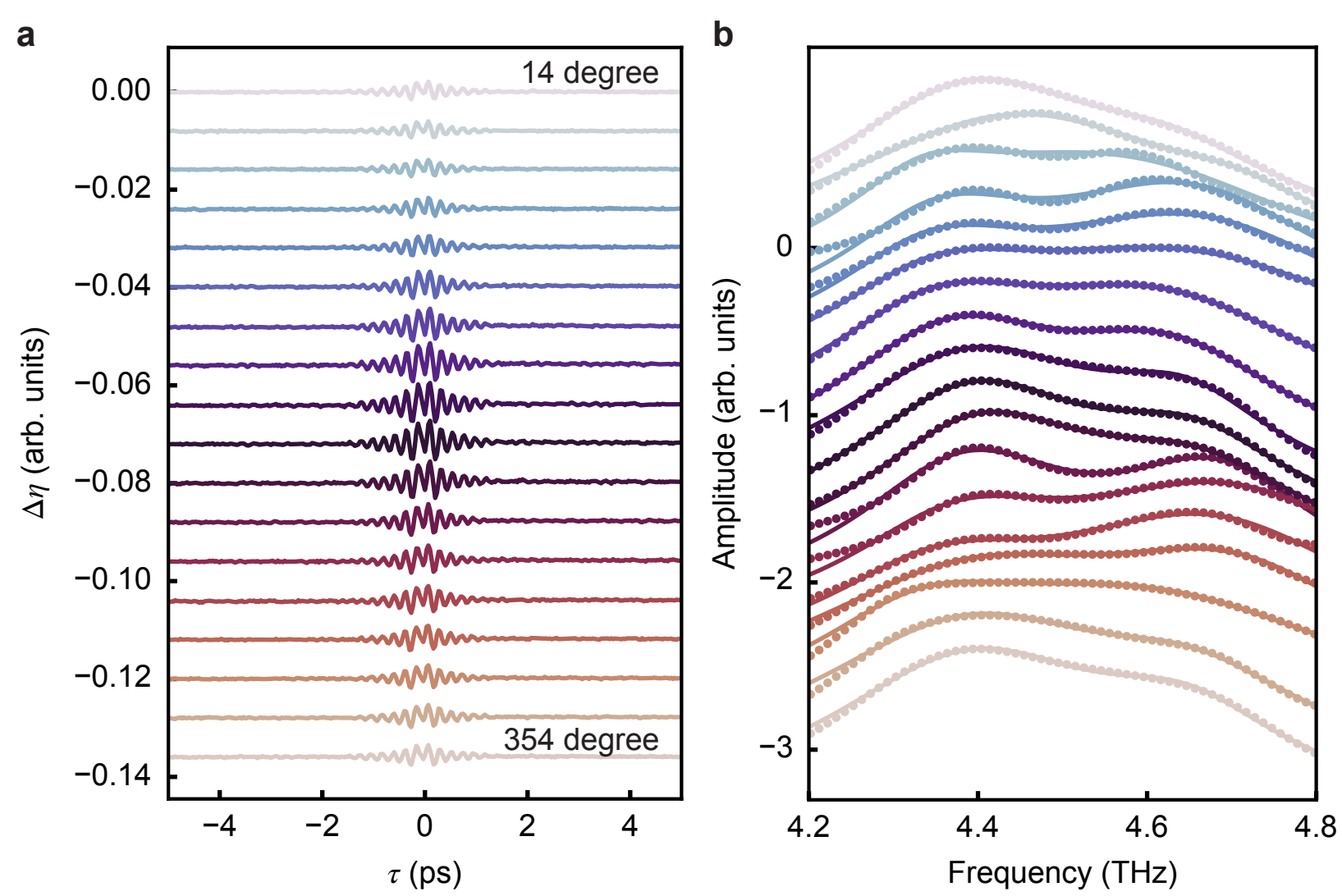

Fig. S2. **THz polarization dependence of the coherent modulation. a,** Coherent modulation amplitude as a function of two-THz delay τ at various THz polarizations. **b,** Fourier transform of **a** and fitting with Eq. 7.

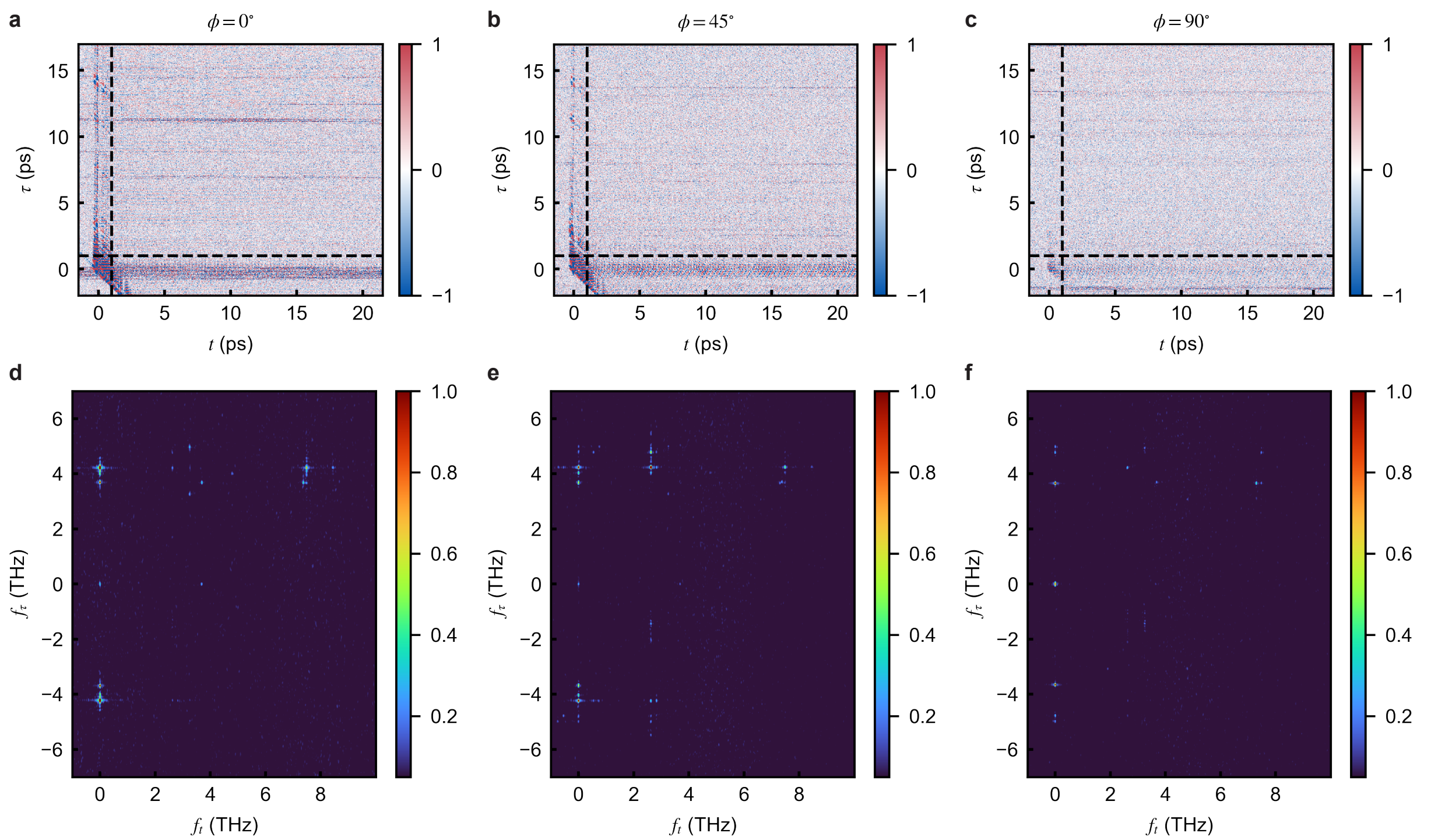

Fig. S3. **2D THz signal as a function of time delays** (t, τ)**. a-c,** 2D THz response measured at THz polarization angles $\phi = 0°$, 45°, and 90°. The black dashed lines demarcate the region used for two-dimensional Fourier transformation analysis. The signal strength is represented in arbitrary units. **d-f,** Fourier transform of the selected regions in **a-c**.

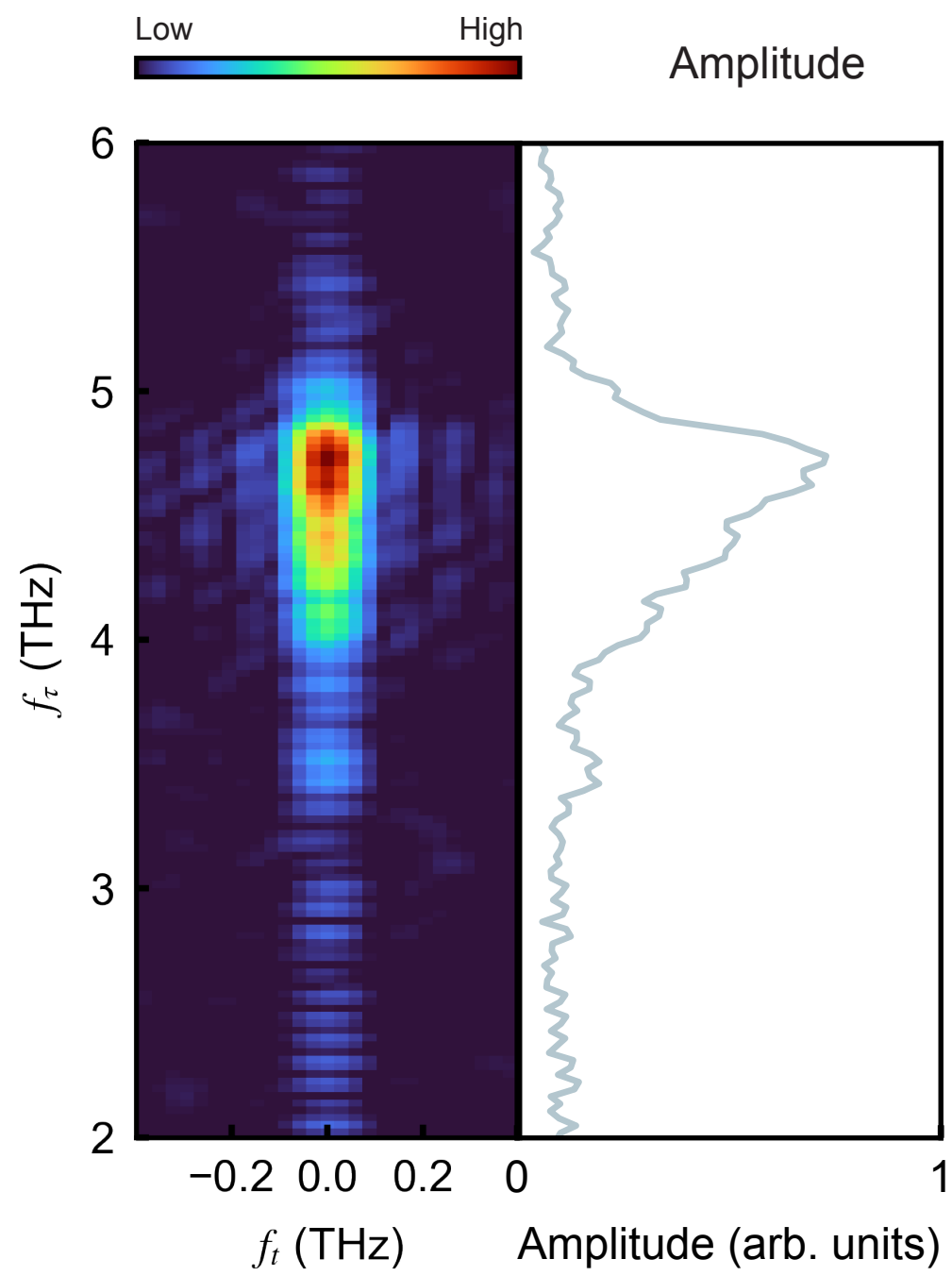

Fig. S4. **2D THz spectrum around** $f_t = 0$ THz **obtained near** T_N**.** Left: 2D THz spectrum near $f_t = 0$ THz obtained at $T = 118$ K. Right: Linecut along $f_\tau = 0$ THz of the 2D THz spectra. The peak corresponding to Q_{IR} exhibits thermal broadening compared to the low temperature results (the blue and red shaded regions in Fig. 3d).

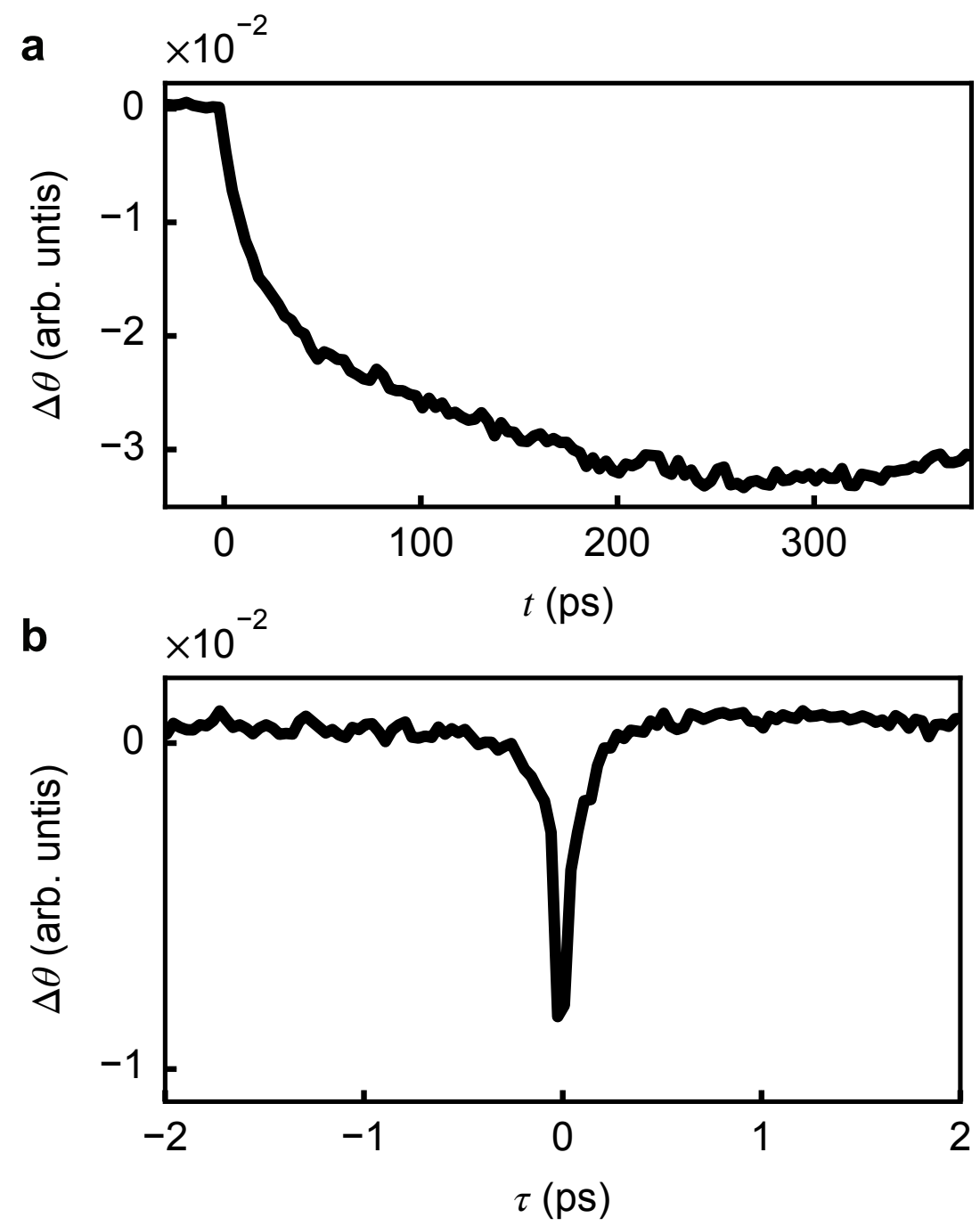

Fig. S5. **Double NIR pump demagnetization response in $FePS_3$ at 115 K. a,** Single NIR pump induced demagnetization as a function of pump-probe delay t. **b,** Double NIR pump modulation of demagnetization as a function of τ measured at $t = 370$ ps.

References

1. J. Lu, *et al.*, Coherent two-dimensional terahertz magnetic resonance spectroscopy of collective spin waves. *Physical review letters* **118** (20), 207204 (2017).

2. B. Ilyas, *et al.*, Terahertz field-induced metastable magnetization near criticality in FePS3. *Nature* **636** (8043), 609–614 (2024).

3. T. Luo, *et al.*, Terahertz control of linear and nonlinear Magno-phononics. *Nature Communications* **16** (1), 6863 (2025).

4. Z. Zhang, *et al.*, Terahertz field-induced nonlinear coupling of two magnon modes in an antiferromagnet. *Nature Physics* **20** (5), 801–806 (2024).

5. E. Viñas Boström, M. H. Michael, C. Eckhardt, A. Rubio, Equilibrium nonlinear phononics by electric field fluctuations of terahertz cavities. *Phys. Rev. Res.* **7**, 033163 (2025), doi:10.1103/fqq1-9f52, `https://link.aps.org/doi/10.1103/fqq1-9f52`.

6. P. C. Hohenberg, B. I. Halperin, Theory of dynamic critical phenomena. *Rev. Mod. Phys.* **49**, 435–479 (1977), doi:10.1103/RevModPhys.49.435, `https://link.aps.org/doi/10.1103/RevModPhys.49.435`.
